# Prevalence of Anxiety Disorder in Adolescents in India: A Systematic Review and Meta-Analysis

**DOI:** 10.7759/cureus.28084

**Published:** 2022-08-16

**Authors:** Debkumar Pal, Dinesh P Sahu, Shampa Maji, Manish Taywade

**Affiliations:** 1 Community Medicine and Family Medicine, All India Institute of Medical Sciences, Bhubaneswar, Bhubaneswar, IND; 2 Pharmacology, All India Institute of Medical Sciences, Bhubaneswar, Bhubaneswar, IND

**Keywords:** india, meta-analysis, systematic review, adolescent, mental health, anxiety

## Abstract

Anxiety is one of the most common mental disorders in the adolescent age group due to both physiological and psychological changes along with substance use in this age group. Generalized anxiety disorder, obsessive-compulsive disorder (OCD), panic disorder, post-traumatic stress disorder (PTSD), and social phobia (or social anxiety disorder) constitute anxiety disorders as per the Diagnostic and Statistical Manual of Mental Disorders Fifth Edition (DSM-5) criteria. In India, the National Mental Health Survey was conducted to estimate the burden of different mental health disorders, but the adolescent age group was not included in that survey.

A comprehensive search strategy was used to find out articles from PubMed and ProQuest, along with a risk of bias assessment using two components of the Quality in Prognosis Studies (QUIPS) tool.

The 13 articles included in the meta-analysis were divided into two groups depending on sampling strategy and outcome measurement. Due to more than 99% heterogeneity, the random effect model is used to find the pooled estimate. The pooled prevalence of anxiety disorder among adolescents in India is found to be 0.41 (CI: 0.14-0.96) for studies with more than low risk and 0.29 (CI: 0.11-0.46) for studies with low risk. The Begg and Mazumdar rank correlation test revealed no publication bias in the included studies. One study was found to be an outlier using the Baujat test, but pooled estimate and heterogeneity did not change significantly after its removal from analysis. The weight of individual studies calculated using the random effect model did not show any gross difference.

A significant burden of anxiety was found in adolescents in India. Effective intervention should be planned to reduce this burden.

## Introduction and background

Anxiety is one kind of emotion where feelings of tension, worried thoughts, and physical changes such as increased blood pressure and pulse rate happen in the presence of any internal or external anticipated danger [1]. Though anxiety is a common phenomenon, sometimes it may be diagnosed as a mental disorder. The anxiety will be called a mental disorder when it persists for a longer duration and affects normal daily life [2]. Generalized anxiety disorder, obsessive-compulsive disorder (OCD), panic disorder, post-traumatic stress disorder (PTSD), and social phobia (or social anxiety disorder) are the five types of mental disorders that come under the anxiety group of mental disorders as per the Diagnostic and Statistical Manual of Mental Disorders, Fifth Edition (DSM-5) [3]. Anxiety disorders are found to be one of the most common mental health disorders in the adolescent age group, i.e., 10-19 years. The adolescent age group is a risk factor for different mental disorders due to psychological and physiological changes [4]. Even substance abuse, which is common in this age group, can cause different types of anxiety disorders [5]. In India, the National Mental Health Survey (NMHS) was conducted in 2015-2016 regarding the burden of different mental health disorders [6]. The NMHS was conducted in different parts of India (North [Punjab and Uttar Pradesh]; South [Tamil Nadu and Kerala]; East [Jharkhand and West Bengal]; West [Rajasthan and Gujarat]; Central [Madhya Pradesh and Chhattisgarh]; and North-East [Assam and Manipur]). But this survey did not include those below 18 years. In India, the adolescent population constitutes 20% of all population nearing to almost 30 crores, which is a very significant part of productivity of the country [7]. Though different studies have mentioned the prevalence and factors related to anxiety disorders among adolescents in India, no systematic review or meta-analysis exists in the literature. The different cross-sectional studies used different tools for diagnosing anxiety along with different types of sampling strategies. We aimed to review systematically the studies published on the prevalence of anxiety in the adolescent age group of India and to estimate the pooled prevalence of anxiety disorders in India.

## Review

Methodology

Eligibility Criteria

All cross-sectional studies published since 1990 where the prevalence of any type of anxiety disorder was estimated were included in the study. We included all the studies where the age group of the sample population belonged to 10-19 years. If more than 50% of the sample belonged to the 10-19 years of age group, then those studies were also included. The studies that reported any type of anxiety disorder such as generalized anxiety disorder, OCD, PTSD, panic disorder, and social phobia (or social anxiety disorder) were included. We excluded all other studies that did not fulfill the inclusion criteria.

Search Strategy

We searched Medline and ProQuest databases for peer-reviewed articles. The search strategy was developed using combined terms related to anxiety, general anxiety, mental health, anxiety disorder, phobia, stress, obsession, panic, India, prevalence, cross-sectional, and burden. From ProQuest, only thesis and dissertations were chosen using the appropriate filter [8]. A detailed search strategy specific to both databases is mentioned in Supplementary 1.

Risk of Bias Assessment

The two dimensions of the Quality in Prognosis Studies (QUIPS) tool that are relevant to observational studies, (1) study participation and (2) study outcome, were used to assess the likelihood of bias in the articles included in the study [9]. Each domain's evaluation yields a subjective estimate of bias risk (low, moderate, or high). The supplementary document provides the tool for risk of bias assessment (Supplementary 2).

Data Extraction

A data extraction sheet was used to extract the data regarding the authors' name, study area, study participants, sampling strategy, age group, and prevalence. Simultaneously, the confidence interval (CI) was calculated and mentioned in the sheet. For most of the studies, the CI value was not mentioned in the original study, and therefore it has been calculated using a formula such as (p̂ +/- z* (p̂(1 - p̂)/n)0.5), where p̂ is prevalence, z value is 1.96, and n is the sample size. The risk of bias was also mentioned in the data extraction sheet.

Reliability

Two reviewers (D.P. and S.M.) checked the articles for the title and abstracted for selection of the studies in a blinded way. Rayyan web-based platform was used for this purpose. In case of any dispute regarding the inclusion of the study, the senior researcher (D.P.S.) took the final decision. All data extracted were checked by all three reviewers.

Analysis

We have provided a descriptive analysis of all the studies included in the meta-analysis. The I2 statistic, for the variance not due to sampling error across studies, was used to analyze heterogeneity between estimates. High heterogeneity is indicated by an I2 value of more than 75%. We included those papers in the meta-analysis where any form of diagnostic tool was used for detecting any type of anxiety illness in teenagers aged 10 to 19 years, as well as studies with more than half of the participants aged 10 to 19. The meta-analysis was carried out using the R program and a random-effects model (to account for heterogeneity). A 95% C) was derived for a pooled prevalence number. When the estimate for a study went toward either below 20% or above 80% in a meta-analysis of prevalence, log transformation was required for normalization of the distribution of prevalence of all studies. After log transformation, the final pooled result and 95% CIs were back-transformed for the final result. We used the Baujat test to find the study resulting in heterogeneity, and the outlier was removed once to find out the effect of the study in heterogeneity and pooled estimate. We used subgroup analysis on the basis of risk of bias, where we classified the studies having a high and moderate risk of bias and studies having a low risk of bias. We used Meta-Essentials for subgroup analysis.

Ethical issues

As this study analyzed data from studies available in the public domain, no ethical clearance was sought. This systematic review and meta-analysis was registered in PROSPERO before the initiation of the review (reference number: CRD42022345574).

Results

**Figure 1:**
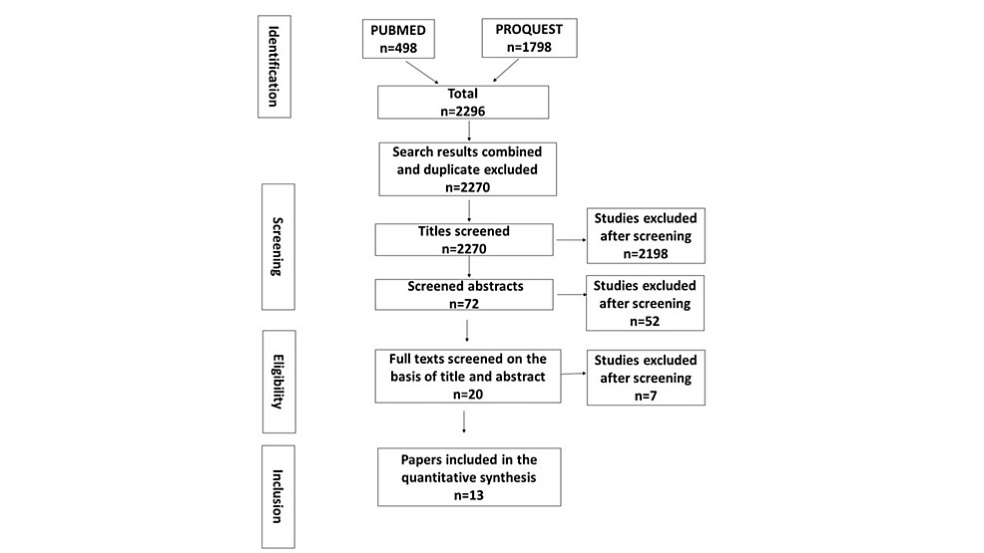
Flow chart illustrating the process by which articles were selected or rejected for inclusion in the study

The search results returned a total of 2,296 articles from the two databases, and after exclusion of duplicates, 2,270 articles were considered for screening by titles. After screening for the titles, 72 articles were selected for screening by abstract. Among full-text screening for 20 articles, finally, 13 articles were selected for quantitative analysis (Figure 1). Two of the articles were excluded for being part of the same study, and five articles were excluded for being review articles.

Included Studies

All of the included studies had a cross-sectional design. Three of the studies used the Screen for Child Anxiety Related Disorders (SCARD) tool [10-12]. DSM-5 and DSM-5 Text Revision (DSM-5 TR) were used in five studies [13-17]. The Depression, Anxiety and Stress Scale - 21 (DASS-21), Westside Test Anxiety Scale, and Test Anxiety inventory were the other tools used in the studies [18-21] (Table 1). In one study, one pre-tested questionnaire was used for diagnosing anxiety disorder [22].

Risk of Bias

All the studies were classified as high, moderate, and low risk on the basis of subjective assessment of studies using the QUIPS tool [23]. Bias in selecting participants and bias in outcome measurement were assessed for all included studies. One study was found to have a moderate risk of bias, and three studies had a high risk of bias. All of the other studies had a low risk of bias (Table 1).

**Table 1:** Description of the studies along with risk of bias assessment DASS, Depression, Anxiety and Stress Scale; DSM, Diagnostic and Statistical Manual of Mental Disorders; MINI, Mini International Neuropsychiatric Interview

Sl no	Author	Study setting	Sampling strategy	Study tool	Age group	Type of anxiety	Sample size	Prevalence	Confidence Interval	Risk of study participation bias	Risk of outcome measurement bias
1	Jayashree et al. [12]	School going children in Mangalore, India	Purposive sampling	Screen for Child Anxiety Related Disorders	15-18 years	Anxiety disorder	201	0.547	0.4757- 0.6174	High	Low
2	Kar and Bastia [13]	School student in cyclone-hit area of Orissa, India	Universal	DSM-IV	11-19 years	Anxiety disorder	108	0.12	0.0657- 0.197	Moderate	Low
3	Kirubasankar et al. [10]	Rural and urban schools in Tamil Nadu, India	Stratified cluster random sampling	Child Anxiety Related Emotional Disorders	14-18 years	Anxiety disorder	462	0.36	0.3155- 0.4049	Low	Low
4	Lohiya et al. [18]	Schools of six Indian states	Multistage stratified random sampling	TAI Questionnaire	9-18 years	Test anxiety	2,158	0.64	0.5655- 0.6056	Low	Low
5	Madasu et al. [11]	Adolescent in rural area, Ballabhgarh, Uttar Pradesh	Random sampling	Screen for Child Anxiety Related Emotional Disorders	10-19 years	Anxiety disorder	729	0.227	0.197- 0.26	Low	Low
6	Mary et al. [19]	Self-financed school of Tamil Nadu	Convenient sampling	Westside Test Anxiety Scale	15-18 years	Test anxiety	100	0.82	0.7305- 0.8897	High	Low
7	Mohapatra et al. [14]	Department of Psychiatry, K. G. Medical University, Lucknow	Convenient sampling	DSM-IV TR	6-16 years	All types of anxiety	1,465	0.0286	0.0207-0.0386	High	Low
8	Pillai et al. [15]	Urban and rural community of Goa	Universal sampling	DSM-IV	12-16 years	Anxiety disorder	2,024	0.0099	0.006- 0.0152	Low	Low
9	Ranasinghe et al. [22]	CBSE schools in India	Cluster random sampling	Structured questionnaire	12-16 years	Anxiety disorder	8,130	0.0761	0.0705- 0.0821	Low	Low
10	Nair et al. [16]	Adolescents of Pattanakkad ICDS block, Allapuzha district in Kerala	Universal sampling	DSM-IV TR	11-19 years	Anxiety disorder	500	0.124	0.0964- 0.1561	Low	Low
11	Sahoo and Khess [20]	Various colleges within Ranchi town	Systematic random sampling	DASS-21 and MINI	17-22 years	Anxiety disorder	405	0.244	0.2034- 0.2893	Low	Low
12	Shaikh et al. [21]	Rural adolescent student of Nanded block, Pune	Purposive sampling	DASS-21	10-19 years	Anxiety disorder	461	0.597	0.5502- 0.6417	High	Low
13	Waghachavare et al. [17]	College students from a rural area of Sangli district, Maharashtra	Stratified random sampling	DSM-IV	17-19 years	Body image anxiety	997	0.427	0.3956- 0.457	Low	Low

Meta-Analysis

**Figure 2:**
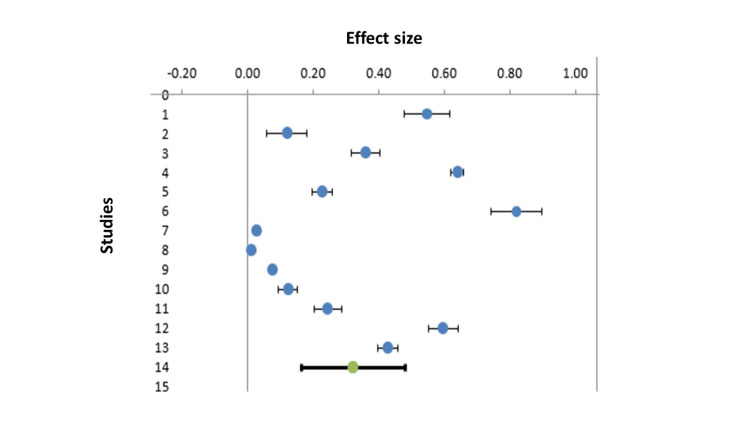
Forrest plot showing pooled estimate

The pooled prevalence was found to be 0.23 with a CI of 0.11-0.41 (Figure 2). The I2 statistics was found to be significant, with a heterogeneity of 99.67%. As the variability was high, random effect model was used to calculate the pooled estimate. During subgroup analysis on the basis of risk bias, the pooled prevalence was found to be 0.41 (CI 0.14-0.96) for studies having more than low risk. The pooled estimate for the studies with low risk of bias is found to be 0.29 (CI 0.11-0.46). Table 2 shows the weightage of different studies with respect to pooled estimates (Table 2). The Baujat test has detected a study conducted by Pillai et al. as an outlier. After removing this study from the analysis, no significant change is detected in heterogeneity and pooled prevalence.

**Table 2:** Weightage of different studies in respect to pooled prevalence using random effect model CI, confidence interval

Sl no	Author	Prevalence	Lower CI	Upper CI	Weightage of studies
1	Jayashree et al. [12]	0.55	0.48	0.62	7.44%
2	Kar and Bastia [13]	0.12	0.06	0.18	7.52%
3	Kirubasankar et al. [10]	0.36	0.32	0.40	7.68%
4	Lohiya et al. [18]	0.64	0.62	0.66	7.81%
5	Madasu et al. [11]	0.23	0.20	0.26	7.76%
6	Mary et al. [19]	0.82	0.74	0.90	7.36%
7	Mohapatra et al. [20]	0.03	0.02	0.04	7.84%
8	Pillai et al. [15]	0.01	0.01	0.01	7.85%
9	Ranasinghe et al. [22]	0.08	0.07	0.08	7.84%
10	Nair et al. [16]	0.12	0.10	0.15	7.77%
11	Sahoo and Khess [20]	0.24	0.20	0.29	7.69%
12	Shaikh et al. [21]	0.60	0.55	0.64	7.67%
13	Waghachavare et al. [22]	0.43	0.40	0.46	7.76%

Publication Bias

**Figure 3:**
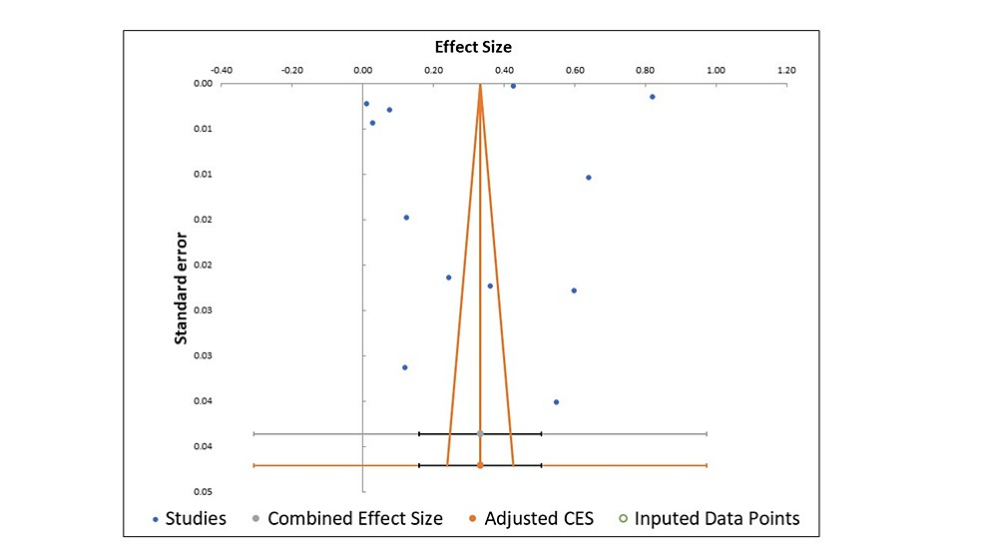
Funnel plot showing publication bias

The Begg and Mazumdar rank correlation test found that the publication bias is not present in this meta-analysis (p=0.085). Figure 3 shows the funnel plot having a symmetrical distribution of studies with respect to standard error and effect size (Figure 3).

Discussion

Out of the 13 studies, nine studies had a low risk of bias and rest of the studies had either moderate or high risk of bias. The pooled estimate for the studies with a low risk of bias was found to be 0.29 (CI: 0.11-0.46) and that for other studies it was 0.41 (CI: 0.14-0.96). The random effect model was used to find out the pooled prevalence as high level of heterogeneity was present among studies. No tool exists for the objective assessment of the quality of bias of cross-sectional studies. Two domains of the QUIPS tool relevant to cross-sectional studies were used here for subjective assessment of bias. This tool was piloted by other authors for the same purpose and was previously used in one meta-analysis [24]. This tool also followed the guidelines of Cochrane collaboration [25]. The prevalence value in different studies can be attributed to different reasons such as type of study population, type of study tool, and type of sampling strategy. Meta-regression analysis could have been conducted to find out those factors. The prevalence of anxiety among adolescents varies in a wide range in different countries. In the USA, approximately 30% of adolescents suffer from some type of anxiety disorder [26]. Among the south-east Asian countries, the prevalence of anxiety in adolescents varies from 21.4% in Pakistan to 9% in Bhutan [27,28]. In the USA, unemployment and substance abuse are found to be significant risk factors for anxiety in adolescents [29]. Poverty and social instability play a crucial role in Pakistan [30]. In Bhutan, the prevalence of substance abuse is found to be lower than that in the USA or Pakistan [29-31]. Those risk factors are prevalent in India also, which lead to similar kind of result in comparison with the USA or Pakistan [32]. This study would help find out the burden of anxiety disorders In India in the pre-COVID-19 era, which has been grossly aggravated due to the COVID-19 pandemic. The COVID-19 pandemic has been found to be a significant risk factor for causing anxiety disorder [33,34].

Strengths

Our study helps get an overview of the burden of anxiety disorders in India, as studies from almost every part of India were included in the analysis. Both types of population such as school students and non-school going children were included in those studies.

Limitations

We did not have access to some databases such as OVID, Embase, Web of Science, and Scopus due to financial constraints. Though we have included two databases as per the requirement prescribed by the Cochrane collaboration group, other databases were not screened.

## Conclusions

This systematic review and meta-analysis has shown that there is a significant burden of anxiety disorders among adolescents in India. As this burden of anxiety disorder can cause significant morbidity in future in the population, effective intervention should be planned to address this. Simultaneously, the burden of other mental disorders should also be estimated in adolescents in India.

## Appendices

Supplementary 1: Search strategy

Search strategy in PubMed:

Population: (((adolescent[Title/Abstract]) OR (adolescence[Title/Abstract])) OR (student[Title/Abstract])) OR (young[Title/Abstract])

Exposure: ((((((((mental health[Title/Abstract]) OR (anxiety[Title/Abstract])) OR (phobia[Title/Abstract])) OR (stress[Title/Abstract])) OR (post-traumatic[Title/Abstract])) OR (anxiety[Title/Abstract])) OR (mental disease[Title/Abstract])) OR (anxiety[MeSH Terms])) AND (India[Title/Abstract])

Location: India

Time period: 1990 to June 30, 2022

ProQuest: anxiety AND India AND Adolescent

Thesis, dissertation, and conference proceeding were selected using filter.

Supplementary 2: Risk of bias assessment [8,22]

**Table 3:** Risk of bias assessment tool

Potential bias	Items to be considered for assessment of potential opportunity for bias
Study participation	
Does the study sample represent the population of interest on key characteristics sufficient to limit potential bias in the results?	Target population: The source population or population of interest is adequately described for key characteristics. Sampling frame: The sampling frame and recruitment are adequately described, possibly including methods to identify the sample (number and type used, e.g., referral patterns in health care), period of recruitment, and place of recruitment (setting and geographic location). The sampling frame and procedures used to sample subjects should not lead to selection of participants that are systematically different from eligible non-participants. Inclusion criteria: Inclusion and exclusion criteria are adequately described (e.g., including explicit diagnostic criteria or “zero time” description). Inclusion/exclusion criteria should not select participants that are systematically different from eligible non-participants. Baseline study population: The baseline study sample (i.e. individuals entering the study) is adequately described for key characteristics. Adequate study participation: There is adequate participation in the study by eligible individuals. Studies should report factors associated with non-response, and quantify and interpret these associations to determine if it is a selective sample. For example, a low participation raises suspicion that there may be a barrier to participating that may influence outcomes.
Outcome measurement	
Is the outcome of interest adequately measured in study participants sufficient to limit potential bias.	Definition of outcome: A clear definition of the outcome of interest is provided, including duration of follow-up and level and extent of the outcome construct. Valid and reliable measure of outcome: The outcome measure and method used are adequately valid and reliable to limit misclassification bias (e.g., may include relevant outside sources of information on measurement properties, as well as characteristics such as blind measurement and confirmation of outcome with valid and reliable test). Measures that are uncommon or have been modified should provide evidence of reliability and validity. Whenever possible, validated instruments should be used. Method and setting of outcome measurement: The method and setting of measurement are the same for all study participants. The measurement approach, timing, and setting of assessment should be standardized across subjects or conducted in a way that limits systematically different measurement. If there are differences, this should be reported and the implications should be considered. Estimation of population parameters: Estimates of population parameters should be calculated using data observed in the whole sample, not extrapolated from rates observed in a subsample (For example, are all participants examined?).

Supplementary 3: PRISMA checklist for this study

**Table 4:** PRISMA checklist

Section and topic	Item #	Checklist item	Location where item is reported
Title			
Title	1	Identify the report as a systematic review.	Page 1
ABSTRACT			
Abstract	2	See the PRISMA 2020 for Abstracts checklist.	Page 1
INTRODUCTION			
Rationale	3	Describe the rationale for the review in the context of existing knowledge.	Page 2
Objectives	4	Provide an explicit statement of the objective(s) or question(s) the review addresses.	Page 3
METHODS			
Eligibility criteria	5	Specify the inclusion and exclusion criteria for the review and how studies were grouped for the syntheses.	Page 3
Information sources	6	Specify all databases, registers, websites, organizations, reference lists, and other sources searched or consulted to identify studies. Specify the date when each source was last searched or consulted.	Page 3
Search strategy	7	Present the full search strategies for all databases, registers, and websites, including any filters and limits used.	Supplementary 1
Selection process	8	Specify the methods used to decide whether a study met the inclusion criteria of the review, including how many reviewers screened each record and each report retrieved, whether they worked independently, and, if applicable, details of automation tools used in the process.	Page 4
Data collection process	9	Specify the methods used to collect data from reports, including how many reviewers collected data from each report, whether they worked independently, any processes for obtaining or confirming data from study investigators, and, if applicable, details of automation tools used in the process.	Page 4
Data items	10a	List and define all outcomes for which data were sought. Specify whether all results that were compatible with each outcome domain in each study were sought (e.g., for all measures, time points, analyses), and, if not, the methods used to decide which results to collect.	Page 5
	10b	List and define all other variables for which data were sought (e.g., participant and intervention characteristics, funding sources). Describe any assumptions made about any missing or unclear information.	Page 5
Study risk of bias assessment	11	Specify the methods used to assess risk of bias in the included studies, including details of the tool(s) used, how many reviewers assessed each study and whether they worked independently, and, if applicable, details of automation tools used in the process.	Page 6
Effect measures	12	Specify for each outcome the effect measure(s) (e.g., risk ratio, mean difference) used in the synthesis or presentation of results.	Page 6
Synthesis methods	13a	Describe the processes used to decide which studies were eligible for each synthesis (e.g., tabulating the study intervention characteristics and comparing against the planned groups for each synthesis (item #5)).	Table
	13b	Describe any methods required to prepare the data for presentation or synthesis, such as handling of missing summary statistics, or data conversions.	NA
	13c	Describe any methods used to tabulate or visually display results of individual studies and syntheses.	NA
	13d	Describe any methods used to synthesize results and provide a rationale for the choice(s). If meta-analysis was performed, describe the model(s) and method(s) to identify the presence and extent of statistical heterogeneity, and software package(s) used.	Page 6
	13e	Describe any methods used to explore possible causes of heterogeneity among study results (e.g., subgroup analysis, meta-regression).	NA
	13f	Describe any sensitivity analyses conducted to assess robustness of the synthesized results.	NA
Reporting bias assessment	14	Describe any methods used to assess risk of bias due to missing results in a synthesis (arising from reporting biases).	Page 6
Certainty assessment	15	Describe any methods used to assess certainty (or confidence) in the body of evidence for an outcome.	NA
RESULTS			
Study selection	16a	Describe the results of the search and selection process, from the number of records identified in the search to the number of studies included in the review, ideally using a flow diagram.	Page 6
	16b	Cite studies that might appear to meet the inclusion criteria, but which were excluded, and explain why they were excluded.	Page 6
Study characteristics	17	Cite each included study and present its characteristics.	Page 7
Risk of bias in studies	18	Present assessments of risk of bias for each included study.	Page 7
Results of individual studies	19	For all outcomes, present for each study: (a) summary statistics for each group (where appropriate) and (b) an effect estimate and its precision (e.g., confidence/credible interval), ideally using structured tables or plots.	Table
Results of syntheses	20a	For each synthesis, briefly summarize the characteristics and risk of bias among contributing studies.	Page 7
	20b	Present results of all statistical syntheses conducted. If meta-analysis was done, present for each the summary estimate and its precision (e.g., confidence/credible interval) and measures of statistical heterogeneity. If comparing groups, describe the direction of the effect.	Page 7
	20c	Present results of all investigations of possible causes of heterogeneity among study results.	NA
	20d	Present results of all sensitivity analyses conducted to assess the robustness of the synthesized results.	NA
Reporting biases	21	Present assessments of risk of bias due to missing results (arising from reporting biases) for each synthesis assessed.	Page 6
Certainty of evidence	22	Present assessments of certainty (or confidence) in the body of evidence for each outcome assessed.	Page 6
DISCUSSION			
Discussion	23a	Provide a general interpretation of the results in the context of other evidence.	Page 7
	23b	Discuss any limitations of the evidence included in the review.	Page 7
	23c	Discuss any limitations of the review processes used.	Page 7
	23d	Discuss implications of the results for practice, policy, and future research.	Page 7
OTHER INFORMATION			
Registration and protocol	24a	Provide registration information for the review, including register name and registration number, or state that the review was not registered.	Page 5
	24b	Indicate where the review protocol can be accessed, or state that a protocol was not prepared.	Page 5
	24c	Describe and explain any amendments to information provided at registration or in the protocol.	NA
Support	25	Describe sources of financial or non-financial support for the review, and the role of the funders or sponsors in the review.	Page 8
Competing interests	26	Declare any competing interests of review authors.	Page 8
Availability of data, code, and other materials	27	Report which of the following are publicly available and where they can be found: template data collection forms, data extracted from included studies, data used for all analyses, analytic code, any other materials used in the review.	Page 8
